# Combined Acetabulum Fracture and Hip Dislocation in an 18-Year-Old Female at 35-Week Gestation: A Case Report and Review of the Literature

**DOI:** 10.1155/2020/8888015

**Published:** 2020-07-23

**Authors:** Joseph R. Young, Lauren Vignaly, Jeremy Carroll, Phillip Ross, Benjamin Villacres Mori, Cory M. Czajka

**Affiliations:** Division of Orthopedic Surgery, Albany Medical Center, Albany, NY, USA

## Abstract

Caring for an injured, pregnant patient can be a management challenge. We report the case of an 18-year-old female who sustained a left acetabulum fracture with a concurrent hip dislocation at 35 weeks' gestation following a motor vehicle accident. Through an interdisciplinary, team-based approach, the patient was guided through obstetric delivery and orthopedic surgical fracture fixation without complication. By being familiar with the unique challenges in management posed by pregnant patients, orthopedic surgeons can be better equipped to minimize morbidity and mortality in this patient population while maximizing clinical outcomes.

## 1. Introduction

The delivery of acute orthopedic trauma care to an injured, pregnant patient is a highly complex endeavor requiring an interdisciplinary, team-based approach. Emergency medicine personnel, orthopedic and trauma surgeons, obstetrician-gynecologists, anesthesiologists, and neonatologists must skillfully address both the mother's injuries and possible injuries to the developing fetus in an effort to prevent significant morbidity and mortality in this subset of patients. Furthermore, it is imperative that providers deliver appropriate care while attempting to minimize future deleterious effects to the developing fetus through the thoughtful and judicious use of ionizing radiation, antibiotics, and anticoagulation.

Pregnant patients that sustain displaced acetabular fractures are a rare subgroup that can oftentimes present a clinical conundrum. Much has been published regarding the successful results achieved with operative fixation of these fractures in nonpregnant patients [1–3]. However, until recently, almost all pregnant patients with acetabular fractures were treated nonoperatively unless the patient's gestational age allowed for safe delivery [1, 4, 5]. This was in part due to concern that the surgery and associated use of radiation presented an unacceptably high risk of injury to the mother and her developing fetus. There are, however, some authors that have contended that operative fixation in these patients may be less morbid than previously thought [1, 6, 7].

We report a case of an 18-year-old female who was involved in an MVA and subsequently sustained a left acetabulum fracture with concomitant hip dislocation as well as a right distal radius fracture. Through a comprehensive, team-based approach, the patient was successfully guided through obstetric delivery and fixation of her acetabular and wrist fractures with successful outcomes experienced by both the mother and her child.

The patient provided informed consent for the publication of the details of this case.

## 2. Case Presentation

An 18-year-old female with a history of psychogenic nonepileptic seizures presented to our Emergency Department (ED) as a Level II trauma activation following a high-speed MVA. Per emergency medicine service (EMS) personnel, the patient was an unrestrained driver traveling approximately 40 miles per hour when she lost control of the vehicle and hit a tree. The patient was hemodynamically stable and conscious on arrival. After obtaining a thorough history, it was determined that the patient was pregnant with a gestational age of 35 weeks and five days. Doppler ultrasound in the trauma bay confirmed that fetal heart sounds were normal.

**Figure 1:**
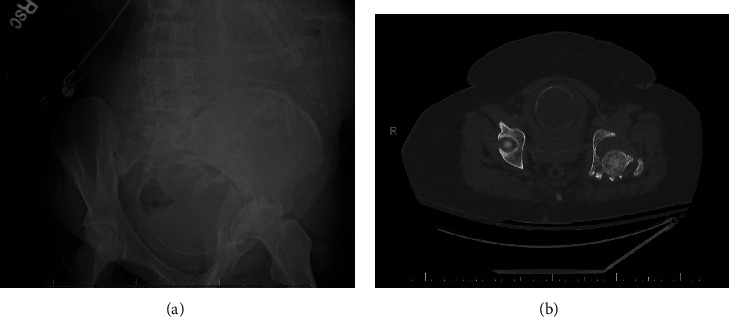
(a) AP radiograph and (b) axial CT scan of the pelvis obtained in the initial trauma evaluation, demonstrating a left transverse posterior wall acetabulum fracture with concurrent hip dislocation in this patient with a gravid uterus.

**Figure 2:**
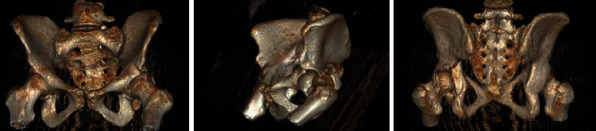
Three-dimensional reconstructions of the CT scan obtained in the initial trauma evaluation, further delineating the fracture pattern.

The patient underwent a primary trauma survey by the trauma surgery team and was determined to be stable. On the secondary survey, the patient complained exclusively of left hip and right wrist pain. Obvious shortening of her left lower extremity was noted. She was otherwise found to be neurovascularly intact. The orthopedic service was immediately consulted. Radiographs were obtained demonstrating a left-sided, displaced transverse posterior wall acetabulum fracture with concomitant hip dislocation as well as a right-sided, volarly displaced distal radius and ulnar styloid fracture (Figures 1 and 2). The patient's wrist fracture was closed reduced and splinted. Reduction of the hip fracture-dislocation was attempted. The position of the hip was improved; however, a postreduction CT scan demonstrated residual dislocation due to instability imparted by a large posterior wall fracture fragment. The patient was placed into skeletal traction at this time.

Shortly after the patient's presentation, the Obstetrics/Gynecology (Ob/Gyn) service was consulted. The patient was evaluated, and the fetus was determined to be in no immediate danger; thus, there was no indication for immediate preterm delivery. Placental abruption was also ruled out with serial fibrinogen levels. The patient underwent extended 24-hour fetal monitoring and daily nonstress testing to continually assess for signs of fetal distress, of which there was none. After consultation between the Orthopedic and Ob/Gyn services, the final treatment plan was devised. The patient was administered with betamethasone for fetal lung maturity beginning on hospital day one. The patient subsequently underwent cesarean section on hospital day five without complication. Successively, the patient was taken for open reduction and internal fixation of her acetabulum fracture two days after delivery, and her wrist was subsequently operated on the following day. Throughout this time, the patient was maintained on enoxaparin for venous thromboembolism (VTE) prophylaxis.

The patient's acetabulum fracture was fixed through a Kocher-Langenbeck approach, with the patient positioned prone on a radiolucent Jackson table. General anesthesia was utilized for the procedure, and prophylactic cefazolin was administered prior to incision. Once exposure was obtained, a Schanz pin was placed into the femur at the level of the lesser trochanter in order to distract the hip joint for irrigation and further removal of debris and clotted blood. The hip was reduced. All contused muscle was debrided, and the fracture edges were meticulously cleaned. Using bone reduction clamps and Kirschner wires, the fracture was anatomically reduced, and the fracture was spanned with two 3.5 mm pelvic reconstruction plates. Following fixation, an intraoperative CT scan and postoperative radiographs were obtained, confirming a near anatomic reduction.

**Figure 3:**
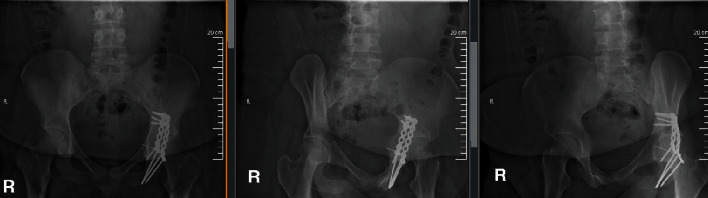
Postoperative AP and Judet radiographs of the pelvis demonstrating stable internal fixation and progression of healing at three months following surgery.

Following fixation of her acetabulum and wrist, the patient continued to be nonweight bearing to her left lower and right upper extremities. She was seen by physical therapy and recommended for rehab placement. She was deemed stable for discharge and transferred to rehab on hospital day 13. The patient was seen in follow-up at three weeks, three months, five months, and one year. She was to remain nonweight bearing for 10-12 weeks. Stable fixation was seen on radiographs at three weeks, with healing apparent at three months (Figure 3). At five months, the patient was ambulating without a limp, and her exam was indistinguishable from that of her uninjured hip. At that time, arrangements were made for her to return to work and gym class. At one year, the patient re-presented for the management of a right lateral malleolus fracture sustained during an unrelated mechanical fall and subsequently underwent open reduction and internal fixation of this injury. Despite this, she continued to have no postoperative complications related to her acetabular fixation. Overall, she has recovered remarkably well without any hip-related complications.

## 3. Discussion

Trauma is the leading cause of nonpregnancy-related maternal death in pregnant patients, and it accounts for 46% of maternal deaths in women under 40 years old in the United States (U.S.) [4, 8–11]. Approximately 6-7% of pregnant patients will have their pregnancies complicated by trauma, with the risk increasing as one's pregnancy progresses [9]. About 55% of these pregnant women are involved in motor vehicle accidents (MVA) [12]. There is an approximately 10-11% maternal mortality rate in those pregnant patients who experience trauma, with the associated fetal mortality rate ranging from approximately 10-15% in the first trimester to as high as 50-54% during the third trimester, mostly due to placental abruption [13, 14]. Approximately 1300 to 3900 pregnancies are lost due to trauma every year in the U.S., with the majority of these losses resulting from minor maternal injuries [15]. 22% of women experiencing orthopedic trauma in particular are multiply injured [16]. Pelvic fractures, in particular, predict a high likelihood of obstetric complications including fetal death [17].

The multiply injured, pregnant orthopedic patient introduces a new set of challenges in management. At all stages of treatment, from prehospital care to postoperative management, the unique physiology of pregnant patients and its associated risks necessitate some modifications to standard trauma protocols.

### 3.1. Relevant Physiologic Changes in the Pregnant Patient and Their Effects on Initial Trauma Evaluation and Management

In order to make informed and effective treatment decisions in this patient population, it is important that all physicians, including orthopedic surgeons, understand the basic physiologic changes that occur during pregnancy, especially regarding a patient's hemodynamic status (Table 1). During pregnancy, the mother's blood volume increases by 30-50%, with a smaller increase in red blood cell mass of approximately 30%, resulting in a dilutional anemia and decreased hematocrit [13, 18]. The implication in this change is that significant hemorrhage of up to 30% of blood may not be readily apparent as the patient is able to adequately compensate. Additionally, the typical signs of hemodynamic instability, such as tachycardia, hypotension, and decreased central venous pressure are oftentimes absent in the second and third trimester [12]. A rapid decrease in the patient's blood volume can decrease uterine blood flow up to 20%, even if the mother is entirely asymptomatic [12]. Therefore, a careful initial trauma evaluation must be completed with a high degree of suspicion when evaluating for hemorrhage, especially in patients with high-energy injuries such as pelvic or acetabulum fractures. Furthermore, all patients admitted for the management of maternal injuries should undergo fetal monitoring for at least 24 hours and may benefit from fetal monitoring throughout the entirety of their hospitalization, even if the mother remains asymptomatic [19]. In our case, the mother was observed very closely, and she underwent close fetal monitoring until delivery despite remaining hemodynamically stable throughout this time.

### 3.2. Radiographic Evaluation

Once the primary survey has concluded and the pregnant patient is deemed to be hemodynamically stable, the secondary survey can commence. In most trauma situations, radiographic evaluation is essential in the diagnosis and management of musculoskeletal injuries. In the setting of trauma, the same tests should be obtained for similar indications as would be ordered in the nonpregnant patient; however, studies should be limited if possible and care should be taken to shield the uterus [9]. There is evidence that this judicious use of imaging is safe and does not result in delayed diagnosis [20]. Despite the concerns expressed by physicians and patients alike with regard to the effects of radiation exposure on the developing fetus, the actual risks are minimal [21]. The currently accepted exposure of ionizing radiation on the fetus is 5 rad, which is significantly higher than any one single diagnostic study (for example, a CT scan of the abdomen and pelvis has a fetal dose of about 2.5 rad) [21–23]. Any exposure above this level portends an increased risk of fetal anomalies or fetal loss [22].

Approximately 30% of the radiation dose received by the mother is taken up by the fetus [9]. The potentially negative effects of radiation on the developing fetus include fetal malformations, alteration of germ-line genes, and induced malignancy [18]. These effects are most present during the period of organogenesis between two and seven weeks' gestation, with the risk decreased to near negligible levels after 20 weeks [9]. If possible, CT of the abdomen and pelvis should be avoided in the first trimester, as it can expose the fetus to an unacceptably high amount of radiation during this critical period of development [9].

### 3.3. Surgical Considerations

Sustaining an acetabulum fracture during pregnancy is a rare event, and the majority of literature examining management of this particular injury is in the form of case reports. While delivery is preferable prior to fracture fixation if the fetus has reached viability, there are instances in which acetabulum fractures have been treated operatively prior to delivery. Yosipovitch et al. reported on the open reduction and internal fixation of an associated both-column acetabulum fracture in a patient at 20 weeks' gestations that was treated via an extended iliofemoral approach [7]. The authors noted that the fracture went on to union, and the patient had an uncomplicated spontaneous vaginal delivery of her child. Charnell et al. reported on a case in which operative fixation of the patient's associated both-column acetabulum fracture was completed immediately following cesarean delivery in the same setting [24]. Here, the authors extended the Pfannenstiel incision into an extended ilioinguinal approach to gain exposure prior to reduction and internal fixation of the fracture with positive results. Almog et al. described two cases in which acetabular fractures were treated conservatively; in one case, the outcome was satisfactory while the second case resulted in posttraumatic arthritis [25]. Additionally, one patient with a displaced acetabular fracture at 20 weeks was treated operatively with no complications to the mother or child [25].

Porter et al. performed a retrospective case series of eight pregnant patients with acetabular fractures treated operatively over a six-year period and compared this group to 518 nonpregnant patients treated surgically for acetabular fractures during this time [1]. Here, the authors reported that all eight patients, ranging from 5 to 26 weeks' gestation, had radiographic signs of fracture healing at 12 weeks. Furthermore, all fetuses reached at least 36 weeks of gestational age, with half being delivered vaginally while the other half were delivered via cesarean section. At the final follow-up, all delivered infants that were evaluated were deemed to be normal and healthy.

The decision to proceed with surgery in pregnant patients with acetabulum fractures is a complex one, and shared decision-making based on a patient specific risk-benefit analysis is paramount. Much of this hesitance is due to a lack of familiarity on the part of orthopedic surgeons with regard to maternal and fetal physiology, as well as the fear that surgical management may harm the mother, the fetus, or both [1]. Additionally, should an adverse outcome occur, it may be difficult to pinpoint whether it was caused by the intervention itself or the normal inherent risks associated with any pregnancy. The aforementioned case studies and small retrospective series published by expert surgeons at academic medical centers demonstrate that it is possible to perform acetabulum fixation in pregnant patients; however, it may not be advisable in the absence of larger scale, prospective data that may be ethically dubious to obtain. For these reasons, surgical management should only occur in pregnancy if absolutely necessary. Once the baby is delivered; however, surgery may proceed according to the regular clearance protocols used in nonpregnant patients. Should emergent surgery be required, the patient should be treated in the same manner as nonpregnant patients, although reasonable accommodations for the fetus can be made so long as they do not cause undue harm to the mother [18]. If surgery is desired after delivery, it is preferable to wait until 39 weeks of gestation to avoid complications associated with preterm births [26]. Should late-preterm (34 0/7-36 6/7 weeks) delivery be deemed necessary, a single dose of antenatal betamethasone is recommended within seven days of delivery to accelerate fetal lung maturity [26].

When considering operative management of pregnant patients with orthopedic injuries specifically, certain considerations with regard to anesthesia and antibiotic usage must be made. General anesthesia has been utilized successfully in previous reports of pregnant patients undergoing acetabulum fixation surgery [1, 7]. While general anesthetic medications do cross the placenta, there is no evidence to suggest that general anesthetic drugs are clearly dangerous to the developing fetus [27]. Despite this, previous evidence has suggested there may be an increased risk of spontaneous abortion associated with general anesthesia in the first or second trimester; however, no exact link has been elucidated [28]. The use of regional or local anesthesia is thus often preferable as a means of decreasing fetal exposure [8]. The decision to use intraoperative fetal monitoring is an individualized one which should be made in consultation with obstetric physicians [29]. When considering antibiotic therapy, it is important to realize that many commonly used antibiotics are contraindicated in pregnancy. Prophylactic administration of cefazolin is recommended for most patients without a *β*-lactam allergy, although clindamycin and vancomycin are acceptable alternatives should an allergy exist [8].

### 3.4. Venous Thromboembolism Prophylaxis

Pregnant women have five times the risk of venous thromboembolism (VTE) compared to their nonpregnant peers, and pulmonary embolism continues to be a leading cause of mortality in this patient population [30]. Thus, great care must be taken to ensure that these women who may be at increased risk are on appropriate prophylaxis. Pregnant patients with inherited or acquired thrombophilias, as well as those immobilized due to trauma, are candidates for thromboprophylaxis. In fact, postoperative pregnant patients have been demonstrated to have an odds ratio of 7.7 for the development of a VTE [31]. Both unfractionated heparin (UFH) and low molecular weight heparins (LMWH) have been safely used in pregnant patients, as neither of these agents crosses the placenta [8]. The American College of Chest Physicians currently recommends the use of LMWH over UFH, due to its favorable side effect profile, more convenient dosing schedule, and lower risk of heparin-induced thrombocytopenia and osteoporosis [32].

## 4. Conclusion

Our case presentation demonstrates many of the challenges inherent in caring for the pregnant orthopedic trauma patient. Through a team-based, interdisciplinary approach, these patients can be successfully guided through delivery and surgical fixation with the expectation of a positive outcome. In our case, the patient and her developing fetus were appropriately diagnosed, resuscitated, and imaged in the ED. The obstetric physicians ensured that the fetus was closely monitored and delivered without complication. The orthopedic team skillfully managed the patient through the perioperative period by providing stable internal fixation and early mobilization, while simultaneously minimizing morbidity with the appropriate use of antibiotics and anticoagulation. Overall, our case demonstrates that excellent results can be achieved in third-trimester patients who sustain acetabular fractures.

## Figures and Tables

**Table 1:** Hemodynamic changes during pregnancy. Reprinted from [13], with permission from Elsevier.

Physiology	Change during normal pregnancy	Normal range during pregnancy
Systolic blood pressure	Decreases by an average of 5-15 mm Hg	110-110 mm Hg
Diastolic blood pressure	Decreases by 5-15 mm Hg	50-70 mm Hg
Mean arterial pressure	Decreases by 10 mm Hg	80 mm Hg
Central venous pressure	Slightly decreases or no change	2-7 mm Hg
Heart rate	Increases by 10-15 beats/min	75-95 beats/min
System vascular resistance	Decreases by 10%-15%	1200-1500 dynes/sec/cm^−5^
Pulmonary vascular resistance	Decreases by 10%-15%	55-110 dynes/sec/cm^−5^
Cardiac output	Increases by 30%-50%	6-7 L/min at rest; 10 L/min with stress
Cardiac index	Increases	4.0-4.5
Pulmonary capillary wedge pressure	Decreases	6-9 mm Hg
Oncotic pressure	Decreases	16-19 mm Hg
Blood volume	Increases by 30%-50%	4500 mL
Red blood cell volume	Increases by 30%	—
Hematocrit	Decreases	32%-34%
White blood cell count	May increase	5000-15000/mm [3]
Electrocardiogram	Flat or inverted T waves in leads III, V_1_, and V_2_; Q waves in leads III and aV_F_	—
